# A field-acquired tea leaf image dataset for disease and pest-damage classification in Bangladesh

**DOI:** 10.1016/j.dib.2026.113356

**Published:** 2026-10-17

**Authors:** Sakibul Hasan Chowdhury, Md Shohel Arman

**Affiliations:** Data Science Lab, Department of Software Engineering, Daffodil International University, Daffodil Smart City, Birulia, Dhaka, 1216, Bangladesh

**Keywords:** Tea leaf disease, *Camellia sinensis*, Image dataset, Computer vision, Precision agriculture, Plant health monitoring, Bangladesh

## Abstract

This data article presents a field-acquired collection of 2500 tea-leaf photographs from four tea-growing locations in Chunarughat, Habiganj, Bangladesh, collected between April 07 and June 23, 2025. The repository contains four image-level categories: Blight (Brown and Gray Blight combined; 555 images), Red Rust (620 images), Helopeltis damage (675 images), and finally Healthy Leaf (650 images). Images were captured under naturally varying illumination, leaf orientation, and background conditions and were retained at their available photographic dimensions. The deposited JPEG images retain their original spatial characteristics, with seven distinct resolutions ranging from 1200 × 1600 to 4608 × 3456 pixels (2411 portrait and 89 landscape images; no square images). The collection was acquired using multiple smartphones and preserves natural field backgrounds rather than standardized laboratory scenes. Manual visual quality screening was used to exclude substantially blurred, unclear, and obvious repeated captures, while no artificial augmentation was applied to the deposited files. The principal contribution of the dataset is the release and documentation of the complete 2500-image field repository for disease- and pest-damage classification; an approximately balanced 2000-image subset was previously used for downstream deep-learning experiments. The complete dataset is openly available through Mendeley Data under a CC BY 4.0 license.

Specifications Table

**Table:** 

Subject	Computer Sciences
Specific subject area	Computer Vision; Precision Agriculture; Tea Leaf Disease and Pest-Damage Classification; Image Dataset; Deep Learning.
Type of data	Images
Data format	Raw/original JPEG images after manual visual quality screening and class organization; no standardized resizing/ cropping or artificial augmentation was applied before deposition.
Data collection	Tea-leaf photographs were acquired between April 7 and June 23, 2025 from four tea gardens/estates in Chunarughat, Habiganj, Bangladesh, using multiple smartphones under natural outdoor conditions. Available EXIF identifies TECNO CK6 (233 images) and OnePlus 11R 5 G (82 images); the authors confirm that the remaining 2185 images were captured using an iPhone 12 Pro Max although make/model EXIF fields are absent in the deposited copies.
Data source location	Chandpur Tea Garden, Deundi Tea Garden, Nalua Tea Estate, and Amo Tea Estate, Chunarughat, Habiganj, Sylhet Division, Bangladesh.
Data accessibility	Repository name: Mendeley Data. Data identification number: 10.17632/3×42rbj8yv.1 Direct URL to data: https://data.mendeley.com/datasets/3×42rbj8yv/1
Related research article	S.H. Chowdhury, M.S. Arman, M.B.H. Siam, M.R. Khan, A. Hasan, P.A.M. Fahim, A curated dataset and lightweight deep learning framework for tea leaf disease classification, PLOS ONE 21(5) (2026) e0349210. DOI: https://doi.org/10.1371/journal.pone.0349210. A 2000-image subset was randomly selected class-wise from the complete 2500 image repository for that study.

## 1 Value of the Data

• The collection provides **2500 original photographs captured in tea gardens**, preserving natural illumination, vegetation, leaf orientation, background clutter, and other field-level variation that can be useful when developing computer-vision systems intended for real agricultural environments.
• Four practically relevant image categories are represented: **Blight (Brown and Gray Blight combined), Red Rust, Helopeltis damage, and Healthy Leaf**, with relatively even class representation ranging from 555 to 675 images per class.
• Researchers can use the images to develop or benchmark image-classification, feature-extraction, transfer-learning, domain-adaptation, and explainable-AI methods without being restricted to images collected against uniform laboratory backgrounds.
• The repository contains **only original, non-augmented images**, allowing users to design their own image resizing, normalization, augmentation, and train/validation/test strategies according to the needs of individual experiments.
• The dataset can support comparative studies of tea-leaf image resources from Bangladesh and other tea-producing regions and can contribute to research on field-oriented crop monitoring and precision agriculture.

## 2 **Background**

Automated recognition of visible plant-health symptoms from digital photographs has become an important application of computer vision in agriculture. Tea plantations present a particularly challenging imaging environment because symptom appearance can vary with disease progression, leaf age, illumination, camera viewpoint, surrounding foliage, environmental conditions, and imaging device. A useful image resource for this purpose should therefore document not only the class labels but also how and where the photographs were collected and what forms of preprocessing were applied before public release.

The public repository comprises 2500 field-acquired images [1]. For the related PLOS ONE modelling study, a 2000-image subset was randomly selected from this already curated collection on a class-wise basis so that the class sizes remained relatively close [2]. The exact filename list used in that experiment was not retained. The present data article therefore documents the complete 2500-image repository, including its acquisition, organization, class composition, technical characteristics, metadata availability, and reuse constraints. In the wider tea-imaging literature, published resources differ substantially in geography, class definitions, acquisition devices, background control, and augmentation strategy, including datasets from China, Bangladesh, and India [[3], [4], [5], [6], [7]].

The four repository labels were designed as image-level plant-health categories. The **Blight** label combines photographs showing Brown Blight and Gray Blight characteristics. Published descriptions of Brown Blight report expanding brown-to-black necrotic lesions on tea leaves [8], whereas Gray Blight is associated with Pestalotiopsis-like fungi and may develop from brown lesions toward gray-centered necrotic areas [9]. Because both symptom groups are intentionally pooled in the present repository, the Blight folder should be interpreted as a combined visual category rather than as a species-level pathogen diagnosis.

The **Red Rust** category represents leaves exhibiting the visually characteristic reddish to orange lesion pattern associated with algal red rust of tea. Recent pathological investigation has associated tea red rust with *Cephaleuros* species [10]. The **Helopeltis** category represents insect-feeding damage rather than a fungal or algal disease. Feeding by Helopeltis theivora can produce puncture marks that gradually darken and develop into necrotic leaf tissue [11]. Retaining Helopeltis as a separate image class therefore enables computational distinction between pest-associated leaf damage and disease-associated leaf symptoms.

The fourth class, **Healthy Leaf**, contains leaves without the visible symptom patterns used to define the three affected categories. Together, these four classes provide a field-image resource spanning healthy leaves, disease-associated symptoms, and insect-related damage.

Recent reviews of plant-disease computer vision and deep learning emphasize that field diversity, sensor differences, background variation, and domain shift can strongly affect model generalization, reinforcing the value of well-documented real-world image datasets [12,13].

## 3 Data Description

### 3.1 Overall composition of the dataset

The released dataset consists of **2500 JPEG files arranged into four class folders**. The class-wise image counts and proportions are given in Table 1.

**Table 1:** Class distribution of the released tea-leaf dataset.

Class	Description	Number of Images	Percentage
Blight	Brown and Gray Blight	555	22.2%
Red Rust	Leaves showing visible Red Rust symptoms	620	24.8%
Helopeltis	Leaves showing helopeltis/ tea mosquito bug feeding damage	675	27.0%
Healthy Leaf	Leaves without visible symptoms represented by the affected classes	650	26.0%
Total		2500	100%

The relatively narrow difference between the smallest class, Blight, and the largest class, Helopeltis, provides a useful starting point for classification research while still preserving the distribution of the curated field collection.

The photographs were acquired at **Chandpur Tea Garden, Deundi Tea Garden, Nalua Tea Estate, and Amo Tea Estate**, all located in Chunarughat, Habiganj, Bangladesh. Representative views of the four collection sites are presented in Fig. 1. Capturing data at multiple tea-growing sites introduced variation in vegetation, surrounding objects, illumination, leaf position, and visual appearance rather than restricting the collection to a single controlled photographic environment.

**Fig. 1:**
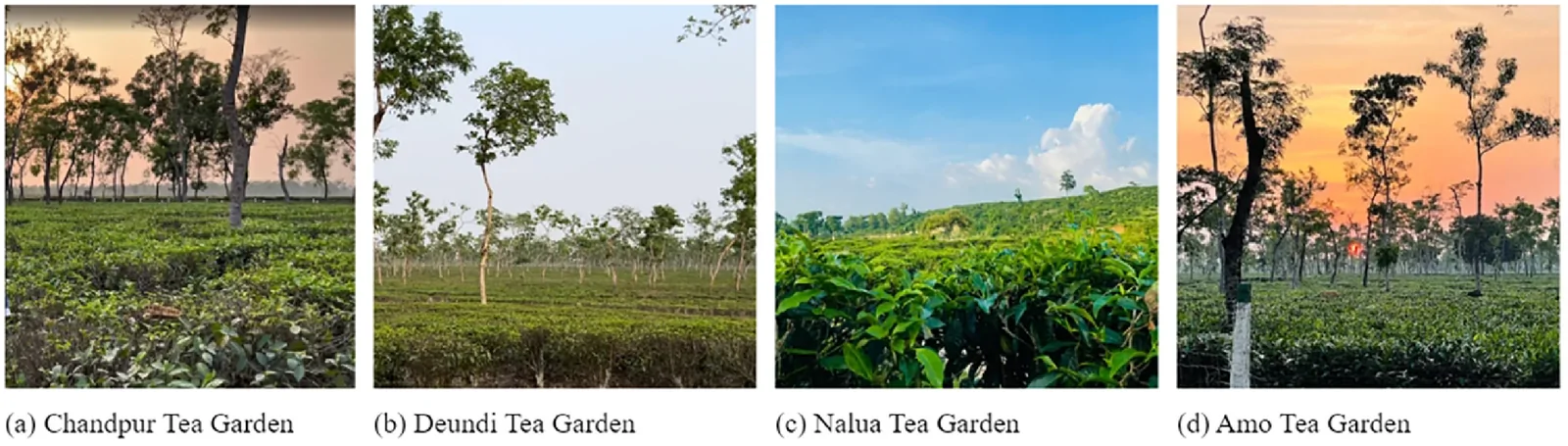
Representative views of the four tea-garden collection locations in Chunarughat, Habiganj, Bangladesh: (a) Chandpur Tea Garden, (b) Deundi Tea Garden, (c) Nalua Tea Estate, and (d) Amo Tea Estate.

### 3.2 Dataset construction workflow

The dataset was constructed through a sequence of field acquisition and curation activities. During collection, individual leaves or tea plants were not assigned specimen identifiers, although the acquisition team attempted to sample across many leaves/plants and to avoid repeatedly photographing the same target leaf. The authors confirm that the field campaign involved well over 50 unique leaf/ plant specimens; however, the exact number cannot be reconstructed retrospectively because specimen-level tracking was not maintained. Images were visually screened before being arranged in class-specific folders for release through Mendeley Data.

This workflow was deliberately kept separate from any model-specific preprocessing. In particular, resizing, normalization, training-time augmentation, and train/validation/test partitioning are **not intrinsic components of the deposited 2500-image collection**. The complete dataset construction and curation workflow is illustrated in Fig. 2.

**Fig. 2:**
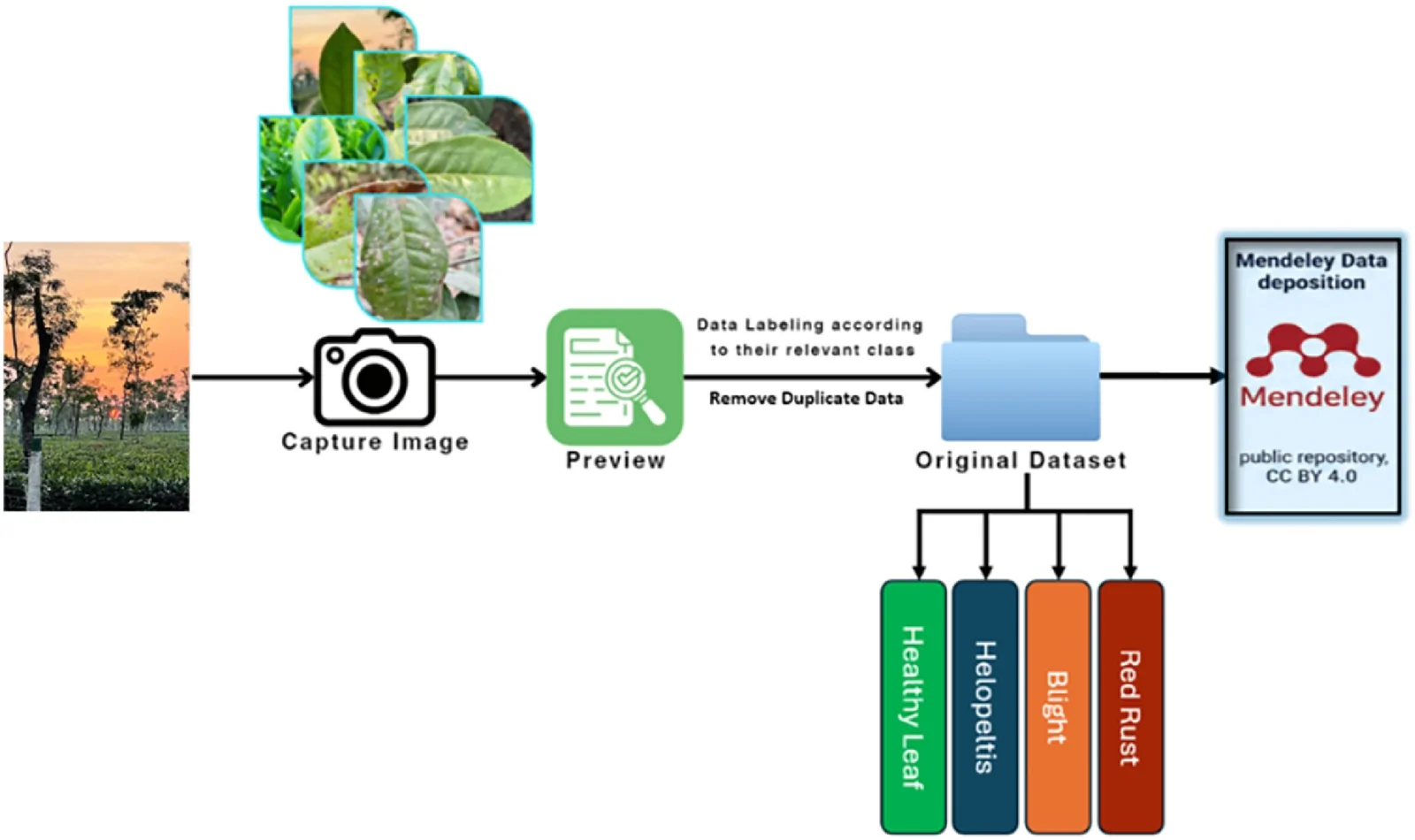
Workflow used to construct the dataset.

#### 3.2.1 Blight

The **Blight** folder contains 555 photographs. Brown Blight and Gray Blight were pooled into a single primary blight class because the dataset was designed for broad image-level recognition of blight associated visual damage rather than separate etiological diagnosis of the two conditions. Filename descriptors such as Brown_Blight and Gray_Blight were retained from the initial visual organization and should not be interpreted as independently laboratory-validated subcategory labels.

Brown Blight commonly produces expanding brown or dark necrotic areas on tea leaves [8]. Gray Blight may also cause necrotic tissue, with affected areas developing a gray center as symptoms progress [9]. Because these symptom types can vary considerably in field images, no pathogen-level subdivision was attempted within the Blight class.

The combined labeling strategy should be considered when designing downstream studies. The class is suitable for systems intended to separate general blight-associated visual damage from Red Rust, Helopeltis damage, and healthy foliage. It should not be interpreted as a dataset specifically designed to discriminate Brown Blight from Gray Blight. Representative examples from the Blight category are shown in Fig. 3.

**Fig. 3:**
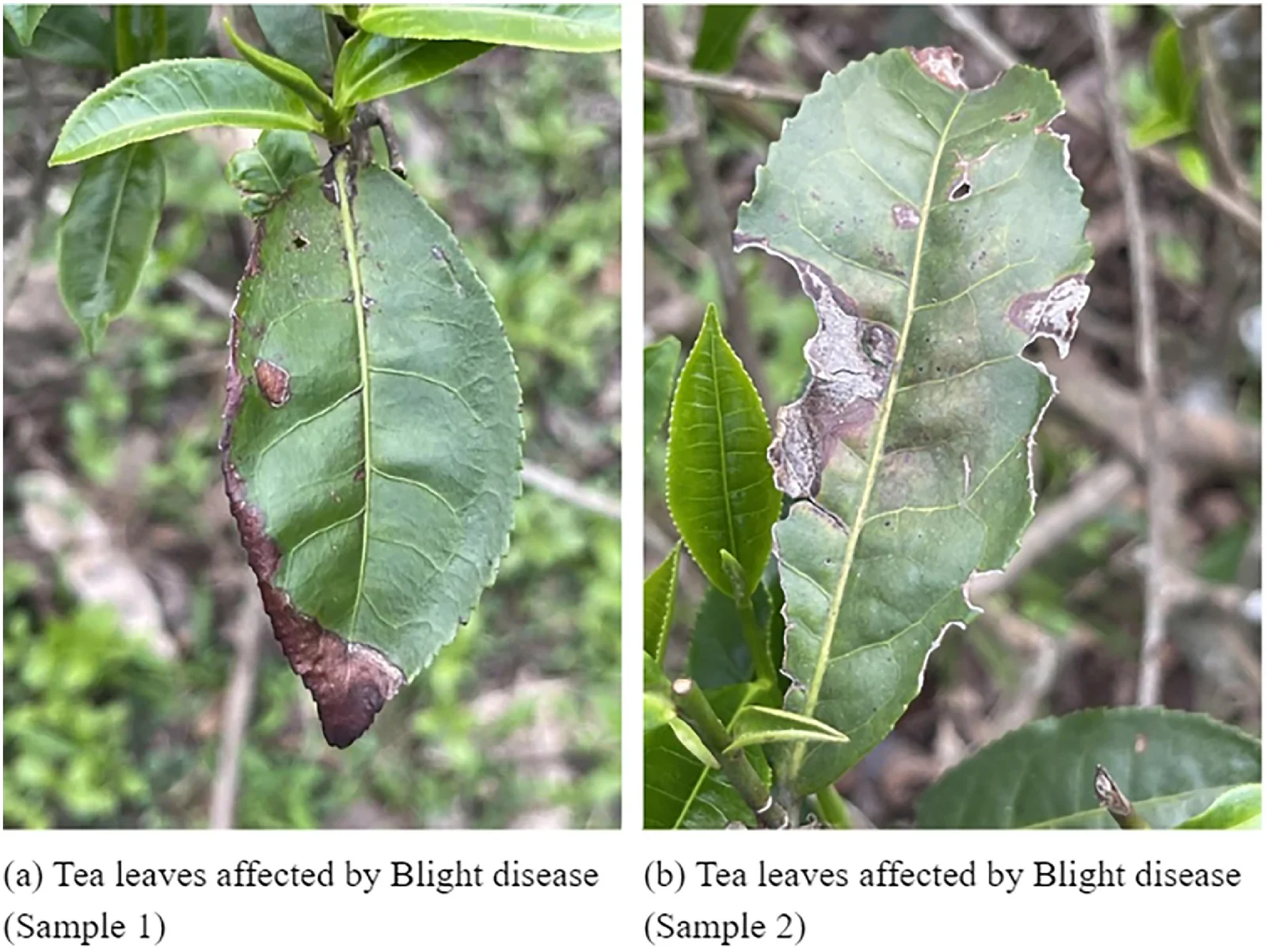
Representative photographs from the Blight class. The class combines images categorized as Brown Blight and Gray Blight.

#### 3.2.2 Red rust

The Red Rust directory contains **620 images**. The visual class is characterized primarily by reddish, orange, or rust-colored affected areas visible on tea-leaf surfaces. Tea red rust is an algal disease associated with *Cephaleuros* species, and recent investigations have demonstrated that more than one *Cephaleuros* species can be associated with red-rust symptoms in tea [10].

For this reason, the repository label is maintained at the **Red Rust symptom-category level** rather than assigning every photograph to a causal species. This distinction is important for reuse: the images are intended for image-level recognition of the visible class represented in the dataset and should not be interpreted as molecular confirmation of an etiological species. Representative Red Rust images are presented in Fig. 4.

**Fig. 4:**
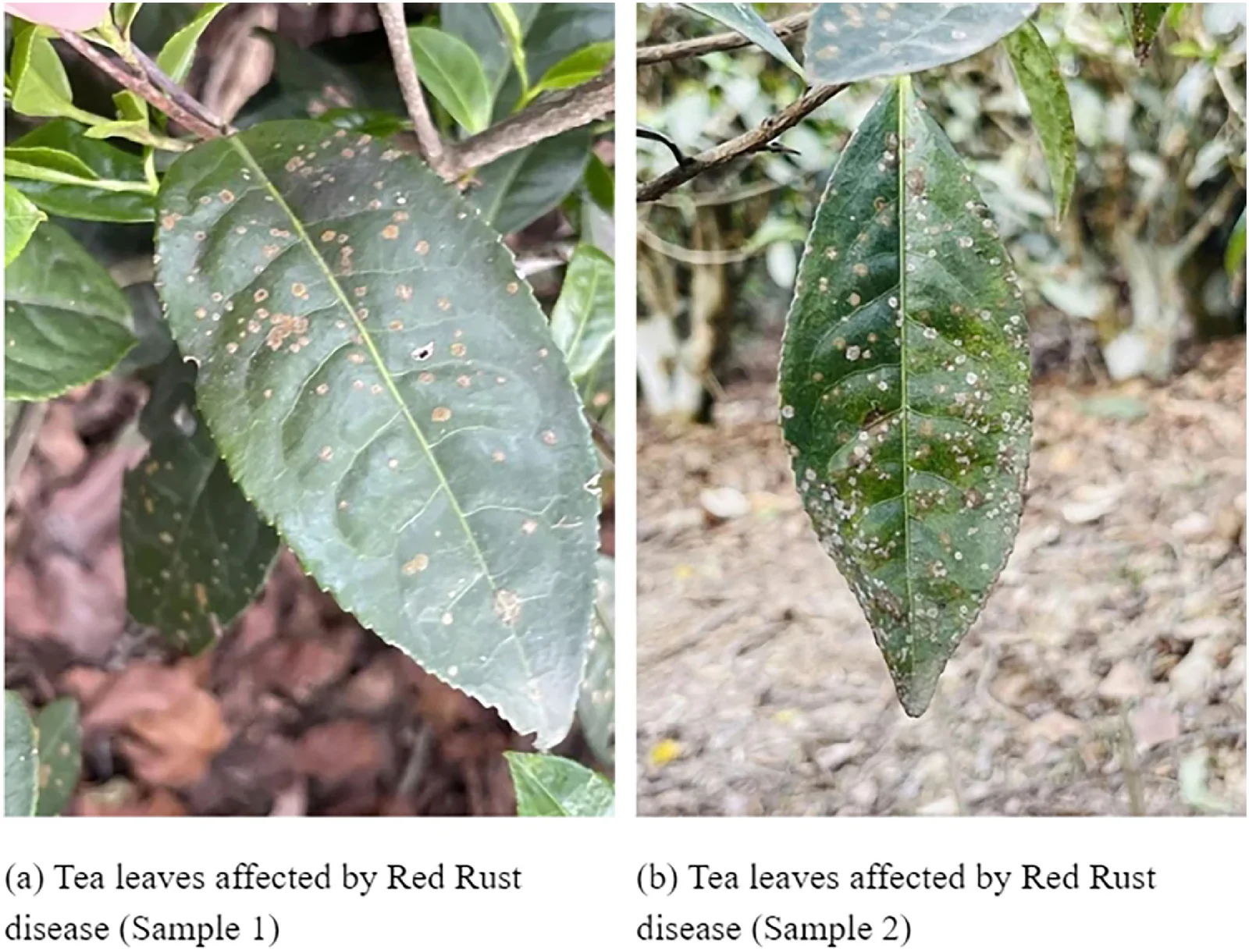
Example images from the Red Rust class showing variation in visible symptoms and field backgrounds.

#### 3.2.3 Helopeltis

The Helopeltis directory contains **675 images**, making it the largest class in the repository. In this manuscript, Helopeltis is treated as a **pest-damage category**, not as a plant disease.

*Helopeltis theivora* is a piercing-sucking tea pest. Experimental characterization of its feeding damage has shown that feeding points can darken after puncturing and that neighboring affected areas can subsequently merge into larger necrotic patches [11]. Consequently, the appearance of Helopeltis-affected leaves can overlap visually with some disease-related necrotic symptoms, making this category useful for image-recognition research where disease and pest injury must be distinguished from one another. Examples of Helopeltis-associated leaf damage are shown in Fig. 5.

**Fig. 5:**
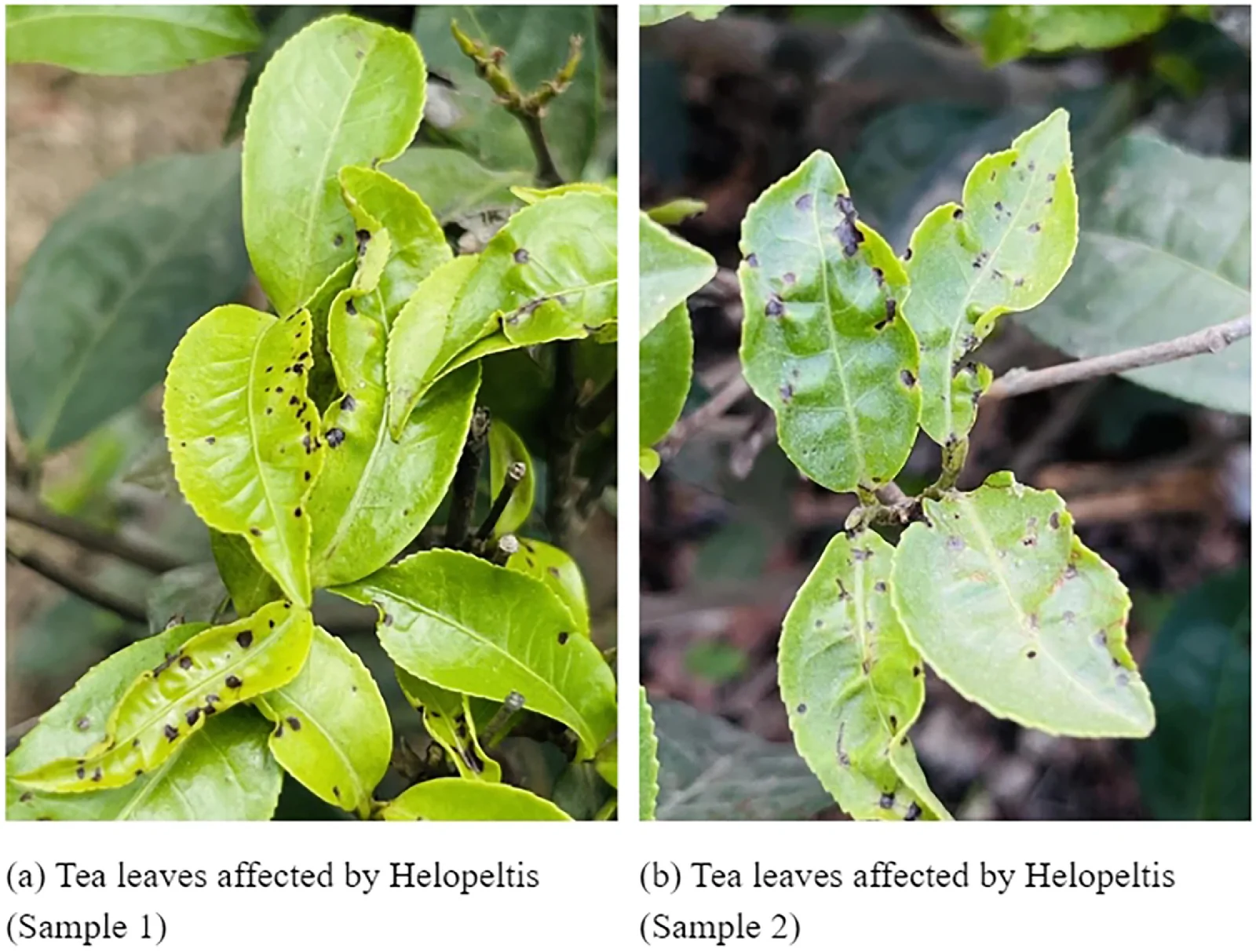
Representative images of tea leaves assigned to the Helopeltis class, showing feeding-related spots and damaged leaf tissue under naturally varying field conditions.

#### 3.2.4 Healthy leaf

The Healthy Leaf class contains **650 images**. These photographs represent tea leaves without the visible lesion, discoloration, or feeding-damage patterns defining the three affected classes in this dataset.

Healthy samples are important because an automated classification system must distinguish affected foliage from visually normal leaves rather than simply separate one affected class from another. Natural variation in leaf size, shape, orientation, illumination, and background was retained within this category rather than enforcing a uniform appearance. Representative Healthy Leaf images are presented in Fig. 6.

**Fig. 6:**
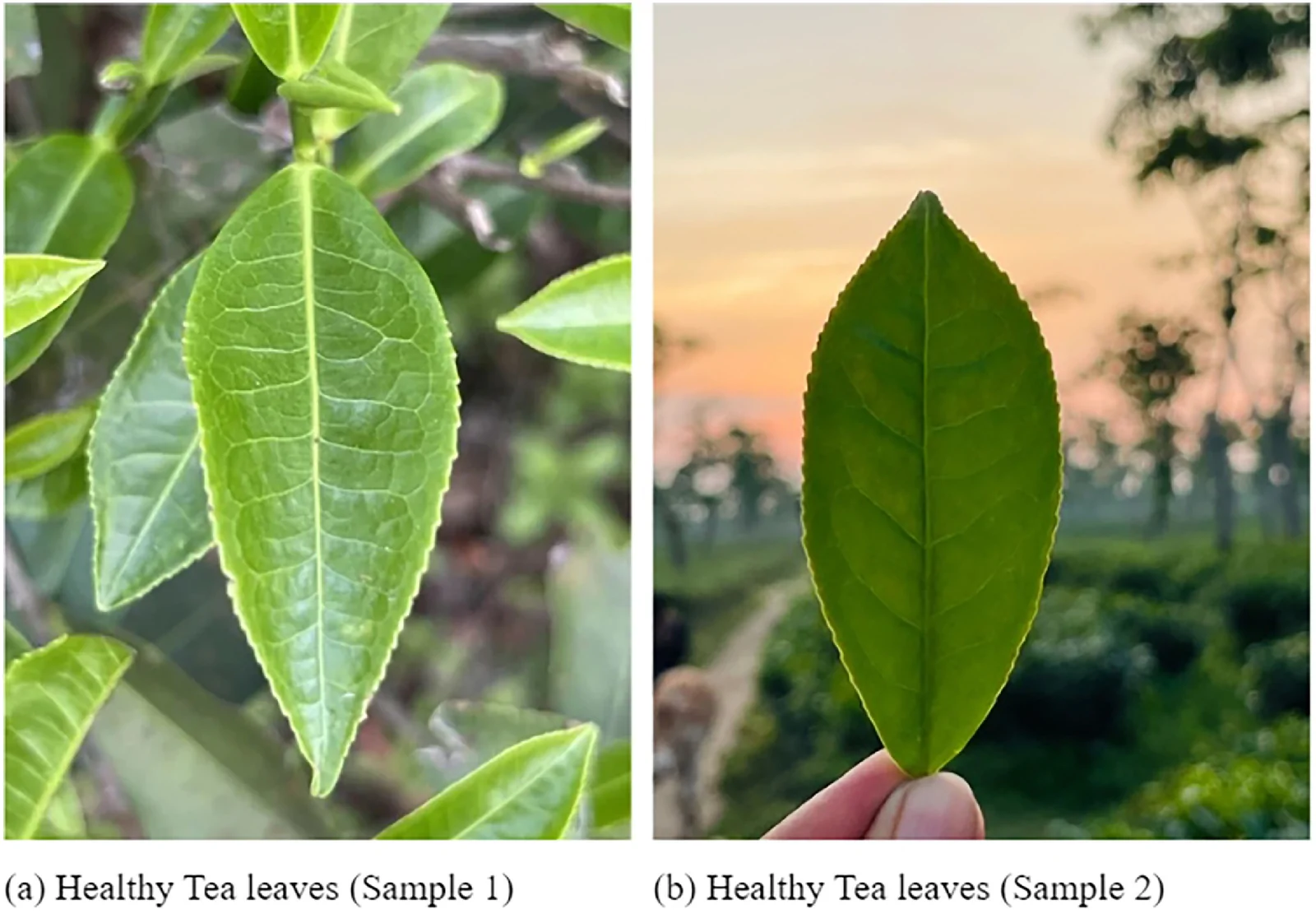
Representative photographs from the Healthy Leaf class, showing naturally occurring variation in leaf orientation, illumination, and field background.

### 3.3 Image and repository characteristics

Table 2 summarizes the principal technical characteristics of the deposited data.

**Table 2:** Technical characteristics of the released dataset.

Attribute	Description
Total number of images	2500
Number of classes	4
Image Classes	Blight, Red Rust, Helopeltis and Healthy Leaf
Blight Definition	Brown and Gray Blight combined
Image format	JPEG (.jpg)
Color representation	RGB photographic images
Resolution range	Seven observed dimension combinations: 1200 × 1600 (2 images), 3072 × 4096 (71), 4096 × 3072 (9), 3024 × 4032 (2146), 4032 × 3024 (39), 3456 × 4608 (192), and 4608 × 3456 pixels (41). Overall: 2411 portrait, 89 landscape, 0 square.
Acquisition device	Multiple smartphones: TECNO CK6 (233 images, EXIF-verified), OnePlus 11R 5 G (82 images, EXIF-verified), and iPhone 12 Pro Max (2185 images, author-confirmed; EXIF unavailable in deposited copies)
Camera system	Smartphone camera systems; model-specific camera metadata are partially available in EXIF.
Acquisition period	April 7, 2025 – June 23, 2025
Number of collection locations	4
Acquisition environment	Natural tea-garden conditions
Approximate per-image file-size range reported in repository	0.67–7.8 MB
Artificial augmentation in deposited dataset	None
Repository	Mendeley Data
Dataset DOI	10.17632/3 × 42rbj8yv.1
Repository version	Version 1
License	CC BY 4.0

An accompanying image-level metadata file is provided with the revised submission. It reports the filename/image ID, primary class label, image dimensions, aspect ratio and orientation, available EXIF acquisition date, and device information. Individual leaf/plant specimen IDs, image-level tea-estate provenance, and image-specific environmental conditions were not systematically recorded during field acquisition and are therefore marked as not tracked or not recorded rather than reconstructed retrospectively. For files whose camera make/ model EXIF fields are absent, the metadata distinguishes author-confirmed device information from EXIF-verified device information. The repository therefore provides the high-resolution photographic files rather than a fixed neural-network input representation. Users remain free to resize the images to dimensions appropriate for a chosen architecture.

#### 3.3.1 Directory organization

The images are stored according to their corresponding visual class. At the conceptual level, the repository follows the directory structure below:

TeaLeaf_Dataset/

│

├—— Blight/

│ └—— [Blight images]

│

├—— Healthy_Leaf/

│ └—— [Healthy leaf images]

│

├—— Red_Rust/

│ └—— [Red Rust images]

│

└—— Helopeltis/

└—— [Helopeltis-damaged leaf images]

This organization permits direct use with image-folder loaders available in commonly used machine-learning frameworks while also making individual classes easy to inspect independently.

#### 3.3.2 Comparison with existing tea-leaf image resources

Several tea-leaf image collections have previously been used or released for automated classification research. Chen et al. developed a field-acquired tea-disease dataset from China containing 3810 original photographs representing seven disease categories and subsequently expanded the working image set through augmentation [3]. Rahman et al. reported 3330 primary images from the Sylhet Division of Bangladesh covering Brown Blight, Gray Blight, Red Rust, and healthy leaves [4]. More recent public Data in Brief resources include a 3960-image tea-garden dataset from Moulvibazar [5], the 5278-image teaLeafBD collection from eight tea gardens in Bangladesh [10], and the CS-D dataset from Assam containing 9000 raw images across six classes [7].

Table 3 compares these resources using **dataset properties rather than model accuracy**. Classification accuracy has deliberately not been used as a dataset-comparison criterion because accuracy depends strongly on model architecture, preprocessing, data partitioning, training protocol, and evaluation design rather than being an inherent property of the image collection.

**Table 3:** Comparison of the present dataset with selected tea-leaf image resources.

Dataset	Region	Original Images	Classes	Acquisition device/ Approach	Image environment	Augmentation status
Present dataset [1]	Chunarughat, Habiganj, Bangladesh	2500	4	TECNO CK6, OnePlus 11R 5 G, and iPhone 12 Pro Max	Natural tea-garden backgrounds	No augmented images in repository
Chen et al. [3]	Hubei, China	3810	7	Canon PowerShot G12	Natural environment	Original collection expanded to 7905 images for analysis
Rahman et al. [4]	Sylhet division, Bangladesh	3330	4	Canon EOS 90D DSLR	Primary field-acquired images	3330-image primary dataset reported
Rimon et al. [5]	Moulvibazar, Bangladesh	3960	4	Vivo V21 and Redmi Note 11	Tea-garden images	Article reports both original and augmented data
Alam et al. (teaLeafBD) [6]	Sylhet and Sreemangal, Bangladesh	5278	7	Four Smartphone models	Collected leaves photographed against a white background	Repository images reported as raw/ original
Gupta et al. (CS-D) [7]	Assam, India	9000	6	Samsung Galaxy F15 5 G, iPhone 13, iPhone 16	Leaves photographed against a plain background	9000 raw images expanded to 81,000

The comparison shows that publicly documented tea datasets differ substantially in acquisition design as well as class scope. Some prioritize a larger number of disease or pest categories, while others standardize image backgrounds or provide augmented derivatives. The present collection contributes a different combination of characteristics: four classes with comparatively even counts, four collection sites, smartphone acquisition, preservation of natural field backgrounds, and release of the original photographs without an artificially expanded image set.

The figures for the comparison above were checked against the source publications: Chen et al. report 3810 original field images and 7905 after augmentation [3]; Rahman et al. report 3330 primary images [4]; Rimon et al. report 3960 original images [5]; teaLeafBD reports 5278 raw images from seven classes [6]; and Gupta et al. [7] initially collected 9000 images and subsequently expanded the dataset to 81,000 samples through augmentation.

## 4 Experimental Design, Materials and Methods

### 4.1 Study area and collection period

Image acquisition was conducted from **April 7 to June 23, 2025** in tea-growing areas of Chunarughat Upazila, Habiganj, Bangladesh. Four locations contributed to the collection: Chandpur Tea Garden, Deundi Tea Garden, Nalua Tea Estate, and Amo Tea Estate.

The use of several collection sites was intended to avoid limiting the repository to the appearance of a single garden. Differences among locations naturally introduced variation in vegetation density, background composition, illumination, and the appearance of individual tea leaves.

Field photography was carried out under naturally occurring outdoor conditions. Consequently, images include visual variability associated with bright sunlight, cloud cover, humid conditions, shadows, partially illuminated leaves, neighboring foliage, soil, branches, and other background components typical of a tea-garden environment. These characteristics were retained when the target leaf remained sufficiently visible for class assignment.

### 4.2 Image acquisition

Images were captured using multiple smartphones. A complete audit of the 2500 deposited files recovered camera make/model EXIF information for 315 images: 233 were identified as TECNO CK6 and 82 as OnePlus 11R 5 G. The authors confirm that the remaining 2185 images were captured using an iPhone 12 Pro Max; however, the deposited copies do not retain recoverable camera make/model fields for those files. The exact reason for the missing EXIF device fields cannot be established retrospectively, so the revised metadata distinguishes EXIF-verified from author-confirmed device information. A complete dimension audit identified seven width-height combinations: 1200 × 1600 (2 images), 3072 × 4096 (71), 4096 × 3072 (9), 3024 × 4032 (2146), 4032 × 3024 (39), 3456 × 4608 (192), and 4608 × 3456 pixels (41). Overall, 2411 images are portrait-oriented and 89 are landscape-oriented; no square images are present.

Photographs were acquired under natural plantation conditions using the standard smartphone camera view, typically at 1× or 2× zoom depending on the visibility of the target leaf and its symptoms. The exact camera-to-leaf distance was not systematically recorded. The collection includes leaves photographed while still attached to tea plants as well as leaves photographed while being held by hand. Each photograph was intentionally composed around a single target leaf as the primary focal subject; surrounding leaves, branches, soil, or other vegetation may remain visible in the background. Multiple photographs of the same leaf were not intentionally retained.

No standardized resizing or cropping was applied by the authors to homogenize the images before deposition. The repository therefore preserves the available photographic dimensions listed in Table 2. Researchers may resize the photographs, for example to 224 × 224 pixels, during their own preprocessing according to the requirements of the selected model.

### 4.3 Class assignment and quality screening

After collection, the images were organized into four visual categories: **Blight, Red Rust, Helopeltis, and Healthy Leaf**, with Brown and Gray Blight combined under the Blight class. One agricultural domain expert, serving as an Upazila Agriculture Officer under the Department of Agricultural Extension (DAE), Chunarughat, Bangladesh, supported the label-validation process. Before folder assignment, the expert explained the visible phenotypic criteria used to distinguish the categories. The author organized the images into class-specific folders, after which the expert reviewed all folders. When an assignment was considered inconsistent with the visible characteristics, the image was reassigned to the appropriate class following the expert review.

Quality screening was performed manually through visual inspection; no automated duplicate-detection algorithm was used. Substantially blurred or unclear images, obvious repeated captures recognized as duplicates, and images with inconsistent class assignments were excluded or corrected during curation. Multiple photographs recognized as representing the same leaf were treated as duplicates and were not intentionally retained. Approximately 30–35 images (about 1.2–1.4% of the pre-curation image pool) were excluded for blur, lack of clarity, or visually identified duplication; however, a detailed reason-specific exclusion log was not maintained, so exact counts for each exclusion category are unavailable. After curation, the dataset contained 555 Blight, 620 Red Rust, 675 Helopeltis, and 650 Healthy Leaf images, totaling 2500 images.

## 5 Preservation of Original Data and Absence of Dataset Augmentation

Artificially generated variants are **not included** among the 2500 repository images. Operations such as random rotation, flipping, zooming, brightness modification, contrast modification, or other training-time transformations were not used to inflate the number of deposited samples.

This is an important distinction between the data resource and subsequent machine-learning experiments. Researchers may choose to apply augmentation after creating their own training partition, but augmented outputs should not be counted as new original observations from the tea gardens.

Likewise, users may resize the photographs to dimensions required by a selected neural network. Resizing an image to 224 × 224 or another standardized input dimension should be described as part of that user's computational pipeline rather than as a property of the dataset itself.

The repository metadata identifies the released collection as 2500 JPEG images and confirms that artificial augmentation was not applied to the deposited data [1]. The revised manuscript and accompanying metadata file additionally document the complete set of observed image dimensions and the availability of device/date EXIF fields.

### 5.1 Relationship to the previously published PLOS ONE research article

The related PLOS ONE research article used a 2000-image subset selected from the already curated 2500-image collection [2]. Images were removed randomly on a class-wise basis so that the four class sizes remained relatively close; the resulting experimental subset comprised 510 Blight, 475 Red Rust, 490 Helopeltis, and 525 Healthy images. Because the subset was drawn from the same curated pool, it had already undergone the same manual visual quality-screening and label-review process described for the complete repository. A separate file list identifying the exact 2000 filenames used in the PLOS ONE experiments was not retained, so those files cannot now be identified retrospectively with certainty.

That related study then applied its own model-specific preprocessing and training procedures, including resizing, normalization, data partitioning, and training-time augmentation. Those computational procedures should not be confused with the data-generation process documented here. The present Data in Brief article concerns the complete 2500-image repository, and the model-specific resized or augmented inputs generated for the PLOS ONE experiments are not included in the 2500-image count.

The PLOS ONE study therefore represents downstream reuse of a subset of the repository, whereas the present data article documents the full public data resource, including acquisition conditions, image characteristics, metadata availability, class organization, and reuse limitations.

## 6 Suggested Reuse Procedure

For downstream image-classification experiments, researchers can retrieve the four class directories directly from Mendeley Data. The original data should first be preserved unchanged. A working copy can then be resized according to model requirements, followed by the desired train/validation/test partitioning. Because specimen-level identifiers were not recorded, users should perform additional visual or similarity-based screening where appropriate and avoid placing obviously related or repeated images across different data partitions.

Where augmentation is required, it is preferable to apply transformations **after dataset partitioning and only to the training samples** so that transformed versions of the same source image do not leak into validation or test sets. Any augmentation strategy should be reported independently from the original dataset size.

Researchers interested in cross-dataset generalization can also use this repository together with other tea-leaf datasets summarized in Table 3. Such experiments could investigate the effect of geographic location, background type, camera system, and disease-class definition on model transferability.

## Limitations

The dataset has several boundaries that should be considered when designing downstream experiments. First, all images originate from tea gardens within Chunarughat, Habiganj, Bangladesh, and acquisition was limited to April 7-June 23, 2025; consequently, geographic, seasonal, and environmental conditions outside this region are not directly represented.

Second, multiple smartphone models were used. Available EXIF metadata identifies TECNO CK6 and OnePlus 11R 5 G images, while the authors identify the remaining files as iPhone 12 Pro Max captures despite the absence of recoverable make/model EXIF fields in those deposited copies. Device usage is not evenly represented across classes, so camera hardware or in-device image-processing characteristics may act as confounding features. Evaluation on images acquired with other devices is therefore recommended.

Third, the repository contains four visual labels rather than an exhaustive representation of every disease, pest, nutrient deficiency, or physiological disorder affecting *Camellia sinensis*. In particular, Brown and Gray Blight are combined within one Blight class. Researchers requiring separate Brown Blight and Gray Blight recognition would therefore need additional annotation or another dataset. In addition, individual leaf/plant specimen IDs and image-level tea-estate provenance were not systematically recorded, limiting specimen-aware or garden-specific partitioning.

Finally, the photographs retain natural backgrounds. Although this is useful for field-oriented computer vision, background information may become a confounding feature if models learn location- or context-specific visual patterns instead of leaf symptoms. The labels are intended for the visible phenotypic categories represented in the repository and should not be treated as species-level pathogen diagnoses solely on the basis of image appearance. Cross-location, cross-season, cross-device, and external-dataset evaluation are therefore recommended when the goal is deployment beyond the collection conditions.

## CRediT Author Statement

**Sakibul Hasan Chowdhury:** Visualization, Conceptualization, Data Curation, Methodology, Validation, Writing – original draft, Writing – review & editing. **Md. Shohel Arman:** Methodology, Validation, Supervision, Writing – review & editing.

## Ethics Statement

The preparation of this dataset did not involve human participants, human-derived data, or experiments on animals. The repository consists exclusively of photographs of tea leaves acquired in agricultural field environments. No personally identifiable information is contained in the released images.

## Data Availability

The complete dataset is publicly available as:

**Tea Leaf Disease Image Dataset for Automated Classification of Tea Garden Diseases**, Mendeley Data, Version 1, DOI: **10.17632/3** **×** **42rbj8yv.1** [1].

The repository contains **2500 original JPEG images** and is distributed under the **Creative Commons Attribution 4.0 International (CC BY 4.0) license**.

## Declaration of Generative AI and AI-Assisted Technologies in the Manuscript Preparation Process

During the preparation of this work, the authors used **OpenAI ChatGPT** to assist with language revision, manuscript organization, and improvement of clarity. The authors reviewed and edited the resulting text, independently verified the scientific content and references, and take full responsibility for the content of the publication.
